# One New Species and Two New Host Records of *Apiospora* from Bamboo and Maize in Northern Thailand with Thirteen New Combinations

**DOI:** 10.3390/life11101071

**Published:** 2021-10-11

**Authors:** Xingguo Tian, Samantha C. Karunarathna, Ausana Mapook, Itthayakorn Promputtha, Jianchu Xu, Danfeng Bao, Saowaluck Tibpromma

**Affiliations:** 1 Centre for Mountain Futures, Kunming Institute of Botany, Kunming 650201, China; 6271105511@lamduan.mfu.ac.th (X.T.); samantha@mail.kib.ac.cn (S.C.K.); jxu@mail.kib.ac.cn (J.X.); 2 School of Food and Pharmaceutical Engineering, Guizhou Institute of Technology, Guiyang 550003, China; 3 Center of Excellence in Fungal Research, Mae Fah Luang University, Chiang Rai 57100, Thailand; ausana.map@mfu.ac.th (A.M.); baodan_feng@cmu.ac.th (D.B.); 4 School of Science, Mae Fah Luang University, Chiang Rai 57100, Thailand; 5 CIFOR-ICRAF China Program, World Agroforestry (ICRAF), Kunming 650201, China; 6 Department of Biology, Faculty of Science, Chiang Mai University, Chiang Mai 50200, Thailand; itthayakorn.p@cmu.ac.th; 7 Environmental Science Research Center, Faculty of Science, Chiang Mai University, Chiang Mai 50200, Thailand; 8 College of Agriculture & Biological Science, Dali University, Dali 671003, China

**Keywords:** one new species, new combinations, new host records, phylogeny, taxonomy

## Abstract

The genus *Apiospora* is known as a cosmopolitan genus, found across various substrates. In this study, four *Apiospora* taxa were obtained from the decaying stems of bamboo and maize in northern Thailand. *Apiospora* collections were compared with known species based on the morphological characteristics and the DNA sequence data of internal transcribed spacer (ITS), the partial large subunit nuclear rDNA (LSU), the translation elongation factor 1-alpha gene (TEF1-α) and beta-tubulins (TUB2). *Apiospora chiangraiense* sp. nov. and two new host records (*Ap. intestini* and *Ap. rasikravindra*) are introduced here based on the morphological characteristics and multi-locus analyses. Additionally, thirteen species previously identified as *Arthrinium* are introduced as new combinations in *Apiospora*, viz., *Ap. acutiapica*, *Ap. bambusicola*, *Ap. biserialis*, *Ap. cordylines*, *Ap. cyclobalanopsidis*, *Ap. euphorbiae*, *Ap. gelatinosa*, *Ap. locuta-pollinis*, *Ap. minutispora*, *Ap. pseudorasikravindrae*, *Ap. septate*, *Ap. setariae* and *Ap. sorghi*.

## 1. Introduction

*Apiospora* was introduced by Saccardo with *Ap. montagnei* as the type species [1]. The genus was reported in both sexual and asexual morphs. The sexual morphs are characterized by multi-locular perithecial stromata with hyaline ascospores surrounded by a thick gelatinous sheath [2,3,4]. The asexual morph of *Apiospora* was characterized by basauxic conidiogenesis, with globose to subglobose conidia, which are usually lenticular in the side view, obovoid and pale brown to brown [2,5,6]. Species of *Apiospora* are similar in morphology, thus it is difficult to distinguish them without molecular phylogenetic data. The size, color and shape of conidia and the morphology of conidiophores (e.g., size, shape and septation) should be used together to better identify them. For example, conidiophores of some species reduce to conidiogenous cells (e.g., *Ap. bambusae*, *Ap. acutiapicum*), while some species have semi-micronematous to macronematous conidiophores (e.g., *Ap. bambusicola*, *Ap. intestini*).

*Apiospora* species have a worldwide distribution and can be found from various hosts [3,7,8,9]. Most *Apiospora* species are associated with plants as endophytes, pathogens or saprobes, especially on bamboo [2,3,10,11]. To date, more than 25 species have been found from bamboo [2,3,10,11]. *Apiospora* species can cause leaf necrosis and twig dieback in the olive tree (*Olea europaea*), leaf edge spot of the peach (*Prunus persica*), blight disease of bamboo (*Schizostachyum*), leaf spot of rosemary (*Salvia rosmarinus*), kernel blight of barley (*Hordeum* *vulgare*) and brown culm streak of *Phyllostachys praecox* [11,12,13,14,15,16,17]. Some species have also been isolated from lichens, air, soil and animal tissues, and a few species are human pathogens which can cause cutaneous infections in humans [9,18,19,20,21,22,23].

The morphological relationships between *Arthrinium* and *Apiospora* have long been debated after Ellis [24], as the morphological characteristics of these two genera are similar and difficult to distinguish based on morphology alone. *Apiospora* was synonymized under *Arthrinium* by Crous et al. [3] as they found that *Apiospora* is the sexual morph of *Arthrinium* and phylogenetic analyses showed that the two genera formed a monophyletic clade. Meanwhile, the phylogenic analyses results from Pintos et al. [25] showed *Arthrinium* forms a monophyletic clade that separates from all other sequences of *Apiospora* and suggested that *Arthrinium s. str*. could actually be phylogenetically different from *Apiospora*, but this is in need of clarification using the phylogeny of additional species before making a conclusive taxonomic decision on the issue. Recently, Pintos and Alvarado [4] showed that *Apiospora* and *Arthrinium* present independent lineages, thus they separate well into two genera.

Morphologically, the conidia of *Apiospora* are more or less rounded in the face view and lenticular in the side view and conidiophores sometimes develop forming acervuli. Whereas the conidia of *Arthrinium* are variously shaped (angular, curved, fusiform, globose, polygonal, navicular) and the conidiophores of some species have thick blackish septa [14]. Ecologically, *Apiospora* species are mostly reported on Poaceae, while *Arthrinium* species commonly occur on Cyperaceae and Juncaceae. Moreover, *Apiospora* has a worldwide distribution, and species in the genus can be found from tropical and subtropical areas to the Mediterranean, temperate and cold regions, while *Arthrinium* species are rarely found from tropical and subtropical habitats. Hence, Pintos and Alvarado [4] considered that genetic, morphological and ecological differences are sufficient to support the taxonomic separation of the two genera, and accordingly, 55 *Arthrinium* species were transferred to *Apiospora* based on the phylogenetic analyses. Presently, 117 records of *Apiospora* are listed in the Index Fungorum [26].

The aims of this study are to determine the phylogenetic placement of the genus *Apiospora* and describe the three taxa that were isolated from maize and bamboo in Chiang Rai province, Thailand. Based on the morphological characteristics and phylogenetic analyses of a combined dataset of the internal transcribed spacer (ITS), the partial large subunit nuclear rDNA (LSU), the translation elongation factor 1-alpha gene (TEF1-α) and beta-tubulins (TUB2), a new species, *Ap. chiangraiense*, as well as two new host records, *Ap. rasikravindrae* and *Ap. intestini,* are introduced. In addition, thirteen species of *Arthrinium* were synonymized under *Apiospora*.

## 2. Materials and Methods

### 2.1. Sample Collection, Isolation and Morphological Characteristic Examination

Fresh specimens of bamboo and maize culms with fungal fruiting bodies were collected from Chiang Rai, Thailand from September–October 2020. Specimens were brought to the laboratory in plastic Ziploc bags for observation. Senanayake et al. [27] were followed for the morphological observations and single-spore isolation. The morphological characteristics were examined under a stereomicroscope (Motic SMZ-171, Wetzlar, Germany). The conidiomata were observed and photographed using a Nikon ECLIPSE Ni-U compound microscope connected to a Nikon camera series DS-Ri2 (New York, United States). The germinating ascospores were transferred aseptically to fresh potato dextrose agar (PDA) media and incubated at room temperature (25 °C) for 2–4 weeks. The morphological characteristics of cultures were checked and recorded after 30–60 days.

The herbarium specimens have been deposited at the herbarium of Mae Fah Luang University (MFLU) and Kunming Institute of Botany (HKAS), while the living cultures have been deposited at Mae Fah Luang University Culture Collection (MFLUCC). The Faces of Fungi and the Index Fungorum numbers are registered as outlined in Jayasiri et al. [28], and the Index Fungorum [26].

### 2.2. DNA Extraction, PCR Amplification and Sequencing

The genomic DNA was extracted from living pure cultures using the Biospin Fungus Genomic DNA extraction Kit (BioFlux, P.R. China) following the manufacturer’s protocol. The internal transcribed spacer (ITS) with the primer pair of ITS4/ITS5 [29], the partial large subunit nuclear rDNA (LSU) with the primer pair of LR0R/LR5 [30], the translation elongation factor 1-alpha gene (TEF1-α) with the primers of EF1-728F/EF-2 [31,32] and the TUB2 with primers of bt2a/bt2b [33] were used to amplify the genes ITS, LSU, TEF1-α and TUB2. The polymerase chain reaction (PCR) was carried out under the following protocol: the final volume of 25 μL consisting of 2 μL of DNA template, 1 μL of each forward and reverse primers, 12.5 μL of 2× FastTaq Premix (mixture of Taq DNA polymerase, dNTPs, and a buffer) and 9.5 μL of deionized water. The PCR thermal cycle program was as follows: for ITS and LSU: initial denaturation at 95 °C for 5 min, then 35 cycles of denaturation at 94 °C for 30 s, annealing at 52 °C for 30 s and extension at 72 °C for 1 min and final extension at 72 °C for 10 min; for TEF1-α: initial denaturation at 94 °C for 5 min, then 35 cycles of denaturation at 94 °C for 1 min, annealing at 56 °C for 1 min and extension at 72 °C for 90 s and final extension at 72 °C for 10 min; for TUB2: initial denaturation at 95 °C for 5 min, then 35 cycles of denaturation at 94 °C for 45 s, annealing at 55 °C for 45 s and extension at 72 °C for 1 min and final extension at 72 °C for 10 min. The PCR products were checked in 1% agarose gels and sent to Tsing Ke Biological Technology (Kunming) Co., China for sequencing. The sequence quality was checked, and the sequences were condensed with SeqMan. The sequences derived in this study were deposited in the GenBank, and the accession numbers were obtained (Table 1).

### 2.3. Phylogenetic Analyses

The sequences generated in this study were subjected to a basic local alignment search tool (BLAST) search in the GenBank to identify closely related *Apiospora* taxa to the taxa obtained in this study. The sequences of *Apiospora* were also obtained from recently published data [4,18,34,35,36,37,38,39]. Consensus sequences were assembled and aligned using BioEdit and MAFFT v.7.110 online program, respectively (http://mafft.cbrc.jp/alignment/server, accessed on 12 August 2021) [40], and manually edited using BioEdit v7.2.3 [41].

The construction of the combined phylogenetic trees was completed using maximum likelihood (ML) and Bayesian inference posterior probabilities (BYPP), with *Sporocadus trimorphus* (CBS 114203) as the outgroup taxon. The models were selected as GTRGAMMA for maximum likelihood, while the best-fit models were selected as GTR + I + G for ITS, LSU and HKY + I + G for TUB2, and TEF1-α for the Bayesian posterior probability analysis. The maximum likelihood (ML) analysis was performed using RAxML-HPC v.8 [42,43] on the XSEDE TeraGrid of the CIPRES Science Gateway (https://www.phylo.org, accessed on 12 August 2021) [44] with a rapid bootstrap analysis, followed by 1000 bootstrap replicates. The final tree was selected amongst the suboptimal trees from each run by comparing the likelihood scores under the GTRGAMMA substitution model. The Bayesian analyses were performed by MrBayes v. 3.2 [45]. Markov chain Monte Carlo (MCMC) was run for 5,000,000 generations, and the trees were sampled every 100th generation. The first 10% of the trees that represented the burn-in phase were discarded, and only the remaining 90% of the trees were used for calculating the posterior probabilities (PP) for the majority rule consensus tree. Phylogenetic trees were visualized with FigTree v. 1.4.2 [46] and modified in Adobe Illustrator CS5 software (Adobe Systems, USA). The newly obtained sequences in this study were deposited in the GenBank.

## 3. Results

### 3.1. Phylogeny

**Figure 1:**
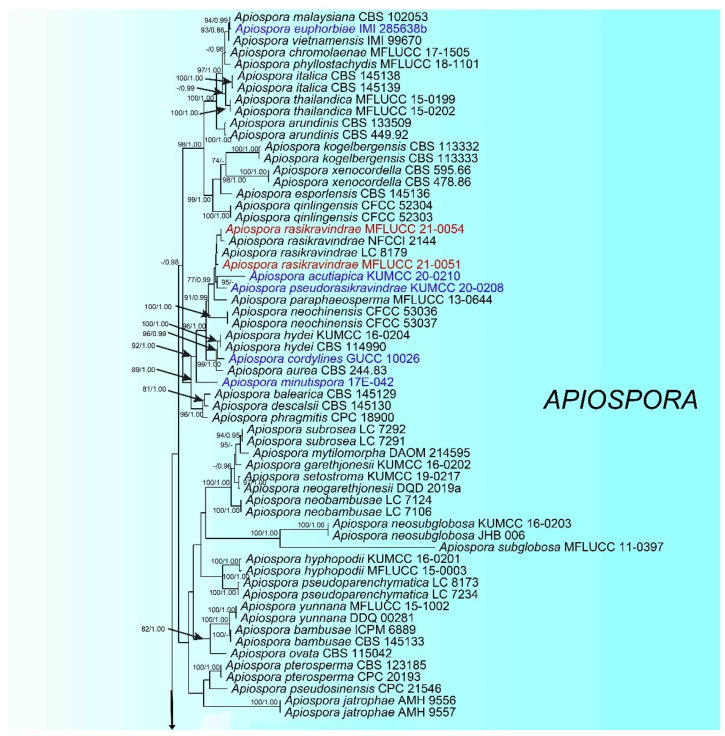

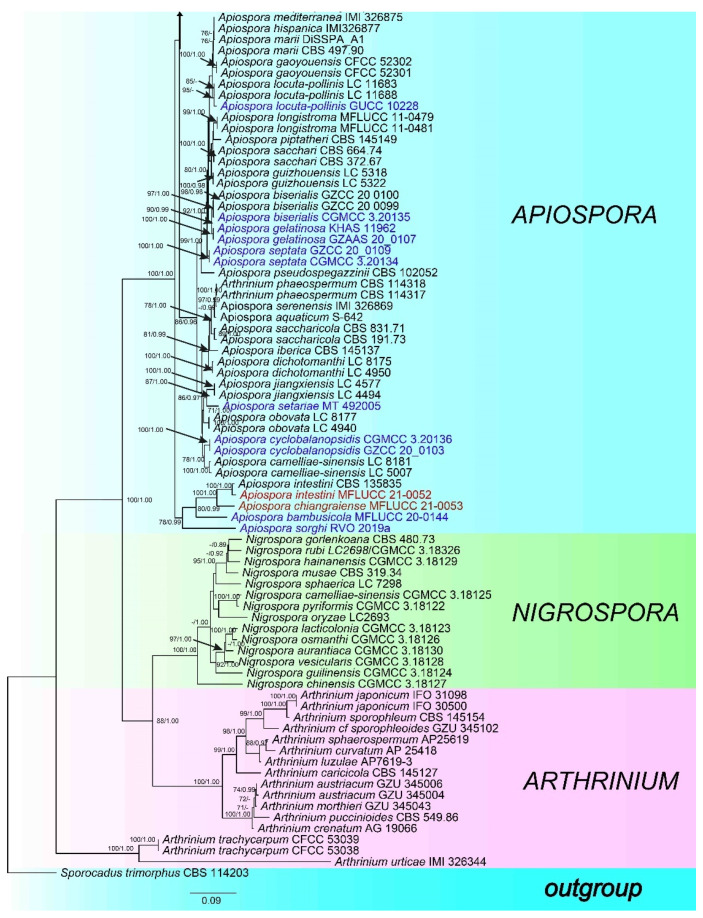
The best-scoring RAxML tree constructed from a concatenated ITS, LSU, TEF1-α, and TUB2 dataset. The tree is rooted with Sporocadus trimorphus (CBS 114203). Nodes were annotated if bootstrap supported value ≥ 70% maximum likelyhood bootstrap proportion (ML, **left**) or ≥0.95 Bayesian posterior probability (PP, **right**). The newly described species are in red and new combination species are in blue.

The combined ITS, LSU, TEF1-α and TUB2 dataset comprised 138 strains, including four newly sequenced strains, with *Sporocadus trimorphus* (CBS 114203) as the outgroup taxon. Multi-locus sequences were concatenated, which comprised 2820 nucleotide characters, including gaps (ITS: 1–637, LSU: 638–1518, TEF1-α: 1519–1971 and TUB2: 1972–2799). The phylogenic tree from the RAxML analysis had similar topology to the Bayesian analysis. The RAxML analysis of the combined dataset yielded the best scoring tree (Figure 1) with a final ML optimization likelihood value of −27840.652840. The matrix had 1446 distinct alignment patterns, with 27.45% undetermined characters or gaps. the estimated base frequencies were as follows: A = 0.238477, C = 0.253732, G = 0.254209, T = 0.253582; substitution rates AC = 1.244445, AG = 3.021293, AT = 1.211434, CG = 1.060781, CT = 4.719948, GT = 1.000000; gamma distribution shape parameter α = 0.298987.

The phylogenetic trees generated by maximum likelihood and Bayesian show the taxonomic placements of our total strains belong to *Apiospora*. The strains MFLUCC 21-0051 and MFLUCC 21-0054 clustered together with members of *Apiospora* and grouped with *Ap. rasikravindrae* (NFCCL 2144 and LC 8179). The strain MFLUCC 21-0052 presented as a distinct lineage and sister to *Ap. intestine* (CBS 135835) with significant statistical support (ML/BI = 100/1.00). The strain MFLUCC 21-0053 clustered with *Ap. intestine* (CBS 135835), but in a distinct clade with high support (ML/BI = 100/1.00).

### 3.2. Taxon Treatment

**Figure 2:**
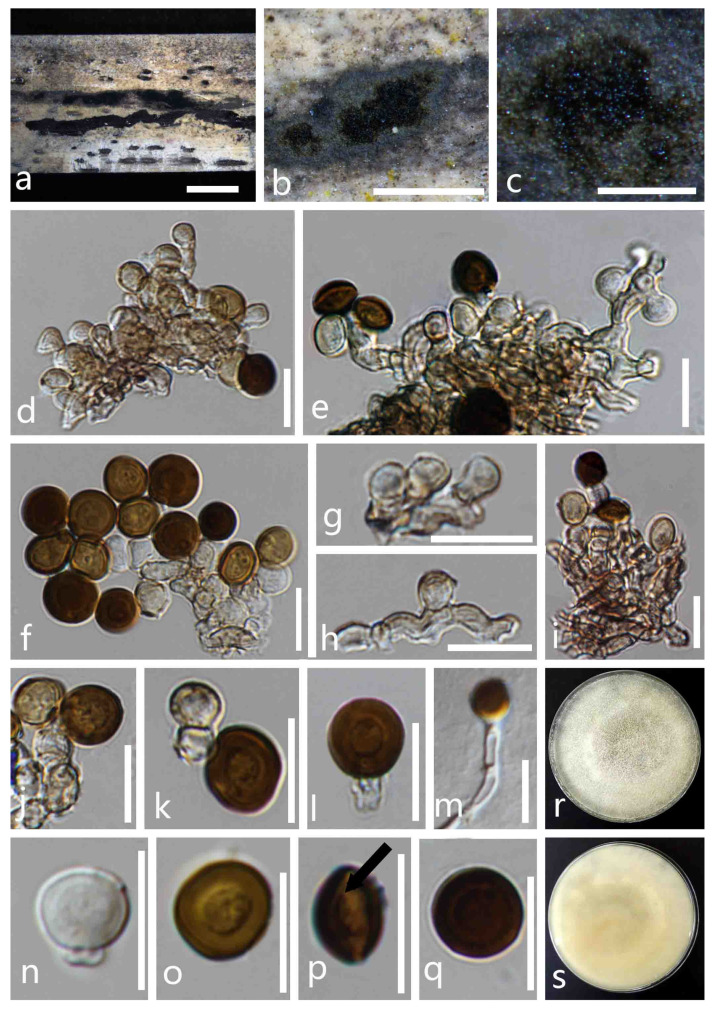
*Apiospora chiangraiense* (MFLU 21-0046, **holotype**). (**a**–**c**) Appearance of the fungus on dead culms of bamboo. (**d**–**i**) Conidia with conidiophores. (**j**–**l**) Conidiogenous cells bearing conidia. (**n**,**o**,**q**) Conidia. (**p**) Conidia with germ-slit. (**m**) Germinated conidium. (**r**,**s**) Colonies on PDA media (**r** forward, **s** reversed). Scale bars: **a** = 4000 μm, **b** = 1000 μm, **c** = 200 μm and **d**–**q** = 10 µm.

*Apiospora chiangraiense* X.G. Tian and *Tibpromma S.*, sp. nov. (Figure 2).

Index Fungorum number: IF558492; Faces of Fungi number: FoF 09905.

Etymology: Referring to Chiang Rai Province, Thailand, where the fungus was collected.

*Saprobic* on dead culms of bamboo. **Sexual morph**: undetermined. **Asexual morph**: *Colonies* on natural substrate are dry, dark brown to black, velvety, dull, consisting of a sterile mycelial outer zone and a round, glistening, abundantly sporulating center, with conidia readily liberated when disturbed. *Mycelium* is superficial, branched, hyaline to dark brown, septate, smooth-walled and hyphae. *Conidiophores* are reduced to conidiogenous cells, grouped together to form sporodochia. *Conidiogenous cells* are 4–7.5 µm high × 3–4 µm diam. ($\overset{-}{x}$ = 6 × 3.5 µm^2^, n = 30), monoblastic or polyblastic, aggregated in clusters on hyphae, hyaline to light brown, smooth and cylindrical to subcylindrical. *Conidia* are aseptate, pale brown to dark brown, in the surface view 6.5–8 × 6–8 μm^2^ ($\overset{-}{x}$ = 7.5 × 7 μm^2^, n = 50), in the lateral view 6–7.5 × 4–5.5 μm^2^ ($\overset{-}{x}$ = 7 × 5 μm^2^, n = 50), with a central scar, globose in the surface view, a lenticular inside view, with straight germ slit spore length.

Culture characteristics: Conidia germinating on PDA within 24 h at room temperature (25 °C). On the PDA, the colonies’ surfaces are white, lightly yellow, wooly, flat, spreading, filiform, with abundant aerial mycelia and reverse off-white to yellow.

Material examined: THAILAND, Chiang Rai Province, Muang District, on dead culms of bamboo, 23 October 2020, X.G. Tian, bb-4-5, (MFLU 21-0046, holotype); ex-type culture, MFLUCC 21-0053.

Notes: In the phylogenetic analyses, *Apiospora* *chiangraiense* formed a distinct clade sister to *Ap. intestini* with strong bootstrap support values (ML/BI = 100/1.00). Morphologically, *Ap. chiangraiense* is distinct from *Ap. intestine* by conidiogenous cells, conidiophores and conidia. The conidiophores of *Ap. intestini* are usually hyaline, macronematous, mononematous and transversely septate, while *Ap. chiangraiense* has reduced conidiophores and conidiogenous cells grouped together to form sporodochia. Additionally, *Ap. chiangraiense* has larger conidia compared to *Ap. intestini* (surface view 6.5–8 × 6–8 μm^2^, lateral view 6–7.5 × 4–5.5 μm versus surface view (4.5–) 5.5 (–6) μm diam, side view (2–) 4 (–6) μm diam). Based on pairwise nucleotide comparisons, *Ap. chiangraiense* is different from *Ap. intestini* (CBS 135835) in 27/583 bp (4.63/) of the ITS, 9/814 (1.1%) of the LSU and 61/696 bp (8.76%) of TUB2, but we were unable to compare TEF-α pairwise nucleotides as the amplification of TEF-α was not successful for this species. However, both the phylogenetic analyses and morphological characteristics supported our species as a distinct new species.

**Figure 3:**
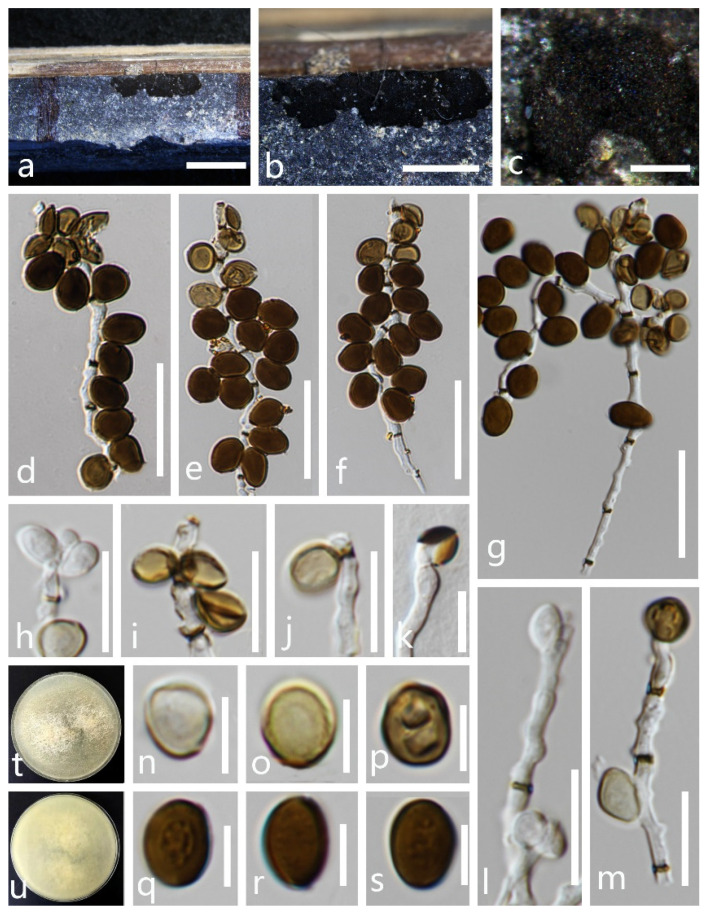
*Apiospora intestini* (MFLU 21-0045). (**a**–**c**) Appearance of the fungus on dead culms of bamboo. (**d**–**g**) Conidia with conidiophores. (**h**–**j**,**l**,**m**) Conidiogenous cells bearing conidia. (**n**–**s**) Conidia. (**k**) Germinated conidium. (**t**,**u**) Colonies on PDA media (**t** forward, **u** reversed). Scale bars: **a** = 4000 μm, **b** = 1000 μm, **c** = 200 μm, **d**–**g** = 20 µm, **h**–**m** = 10 μm and **n**–**s** = 5 μm.

*Apiospora intestini* (Kajale, Sonawane and Rohit Sharma) Pintos and *P. Alvarado*, Fungal Systematics and Evolution 7: 206 (2021) (Figure 3).

Index Fungorum number: IF 837744.

*Saprobic* on dead culms of bamboo. **Sexual morph**: undetermined. **Asexual morph**: *Colonies* are on natural substrate surface, gregarious, powdery, dark brown to black, dull with conidia readily liberated when disturbed. *Conidiophores* are 1.5–2 µm wide ($\overset{-}{x}$ = 2 µm, n = 40) hyaline, macronematous, mononematous, transversely septate, thick-walled, brown. *Conidiogenous cells* are 6–9.5 × 1.5–2 µm^2^ ($\overset{-}{x}$ = 7.5 × 2 µm^2^, n = 30), intercalary, cylindrical, hyaline. *Conidia* are 6.5–5 × 6–10 µm^2^ ($\overset{-}{x}$ = 7 × 5.5 µm^2^, n = 50), borne as bunches on conidiophores, lateral, pale brown to brown, smooth-walled, globose to subglobose or irregularly round, aseptate, with a central scar and without germ slit.

Culture characteristics: Conidia germinating on PDA within 24 h at room temperature. The colonies’ surfaces are white, cottony, flat, spreading, filiform, mycelia not tightly attached to the surface and the reverse lightly pigmented.

Material examined: THAILAND, Chiang Rai Province, Muang District, on dead culms of bamboo, 23 October 2020, X. G. Tian bb-4-2 (MFLU 21-0045), living culture, MFLUCC 21-0052.

Notes: *Apiospora intestini* was introduced by Crous et al. [19] based on the morphology of asexual morph and the phylogenetic analyses. In this paper, our new isolate (MFLUCC 21-0052) clustered with the ex-type strain of *Ap. intestini* with relatively high support (ML/BI = 100/1.00). Morphologically, the conidia of the new isolate (MFLUCC 21-0052) are similar to the holotype *Ap. intestini* (CBS 135835) in having similar size of conidiophores that are borne as bunches, intercalary and terminal, brown, smooth, aseptate and globose to subglobose. Based on nucleotide comparisons, *Ap. intestini* (MFLUCC 21-0052) is slightly different from *Ap. intestini* in 12/580 bp (2.07%) of the ITS, 2/814 (0.24%) of the LSU, 2/684 bp (0.29%) of TUB2 and 2/610 bp (0.32%) of TEF1-α. Based on both phylogeny and morphology, the new isolate (MFLUCC 21-0052) is identified as *Ap. intestini*. This is the first report of *Ap. intestini* (MFLUCC 21-0052) isolated from dead culms of bamboo in Thailand, which was originally isolated from a grasshopper’s gut in India.

**Figure 4:**
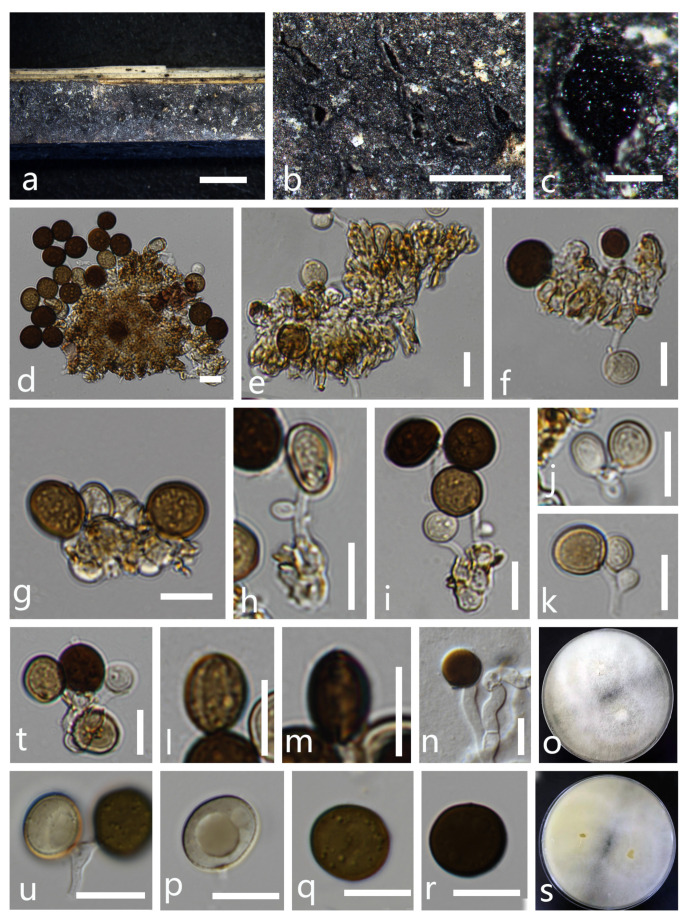
*Apiospora rasikravindrae* (MFLU 21-0045). (**a**–**c**) Appearance of the fungus on dead culms of bamboo. (**d**–**i**) Conidia with conidiophores. (**j**,**k**,**t**,**u**) Conidiogenous cells bearing conidia. (**l**,**m**) Conidia with germ-slit. (**p**–**r**) Conidia. (**n**) Germinated conidium. (**o**,**s**) Colonies on PDA media (**o** forward, **s** reversed). Scale bars: **a** = 4000 μm, **b** = 2000 μm, **c** = 200 μm and **d**–**s** = 10 μm.

*Apiospora rasikravindrae* (Shiv M. Singh, L.S. Yadav, P.N. Singh, Rahul Sharma and S.K. Singh) Pintos and *P. Alvarado*, Fungal Systematics and Evolution 7: 207 (2021) (Figure 4).

Index Fungorum number: IF 837716; Faces of Fungi number: FoF 01994.

*Saprobic* on dead culms of bamboo. *Colonies* appear as spotty patches on natural substrate surface. *Conidiomata* are immersed, pycnidial, scattered, globose to slightly conical, ostiolate, black, coriaceous. *Conidiophores* are 9–26 × 1–2.5 μm^2^ ($\overset{-}{x}$ = 17.5 × 2 μm^2^, n = 15), arising mostly from swollen basal cells, micro to semi-macronematous, mononematous, unbranched, straight or flexuous, smooth and thin-walled, hyaline. *Conidiogenous cells* are basauxic, discrete, hyaline, smooth-walled. *Conidia* in surface view are 9–11 × 9–10.5 μm^2^ ($\overset{-}{x}$ = 10 × 10 μm^2^, n = 50), in lateral view 10–11 × 6.5–8 μm^2^ ($\overset{-}{x}$ = 10.5 × 7.5 μm^2^, n = 20), lenticular, globose to ovoid, occasionally elongated to ellipsoidal, brown to dark brown, smooth-walled, with a longitudinal, thin-walled, with a pale equatorial slit.

Material examined: THAILAND, Chiang Rai Province, Muang District, isolated as *Saprobic* on dead culms of bamboo, 23 October 2020, X. G. Tian, bb-4-1 (MFLU 21-0044), living culture, MFLUCC 21-0051; *ibid* decaying maize culms, 11 November 2020, X. G. Tian, corn-1-1 (HKAS 115764), living culture, MFLUCC 21-0054

Notes: The National Center for Biotechnology Information (NCBI) BLAST results of ITS, LSU, TUB2 and TEF1-α sequences of our new isolate (MFLUCC 21-0054) showed high similarities with *Apiospora rasikravindrae* (LC 8179) (100%, 100%, 98.90% and 98.97%, respectively), while the new isolate (MFLUCC 21-0051) also showed high similarities with *Apiospora rasikravindrae* (LC 8179) (99.83%, 100%, 99.61% and 99.51%, respectively). Our phylogenetic analyses showed that the two new isolates clustered with the ex-type strain of *Ap. rasikravindrae* (NFCCI 2144) and *Ap. rasikravindrae* (LC 8179). Morphologically, our new isolate is closely related to the holotype of *Ap. rasikravindrae* in having lenticular, globose to ovoid, occasionally elongated to ellipsoidal, brown to dark brown, smooth-walled, germ-slit conidia and micro-semi-macronematous, mononematous, unbranched, straight or flexuous, smooth and thin-walled and hyaline conidiophores. Hence, the two new isolates are identified as *Ap. rasikravindrae*.

*Apiospora rasikravindrae* was originally isolated from soil in Norway [47]. *Apiospora rasikravindrae* occurred on *Capsicum*, *Kappaphycus alvarezii*, *Pinus*, *Platanus acerifolia*, rice, *Sargassum thunbergia* and *Triticum aestivum* from Brazil, China, India, Japan, Netherlands, Svalbard and Thailand [3,48]. Dai et al. [3] describe and illustrate both sexual and asexual morphs for *Ap. rasikravindrae* and it was collected on the stems of bamboo. In this study, the isolate MFLUCC 21-0051 was newly collected from bamboo, while the isolate MFLUCC 21-0054 was newly recorded from maize.

### 3.3. New Combinations

*Apiospora acutiapica* (Senan. and Cheew) X.G. Tian and Tibpromma S., comb. nov.

Index Fungorum number: IF558499.

Basionym: Arthrinium acutiapicum Senan. and Cheew, Frontiers in Microbiology 11. 2020.

Notes: *Arthrinium acutiapicum* was introduced by Senanayake et al. [34] and was collected on dead twigs of *Bambusa bambos* in China. Senanayake et al. [34] mentioned that this species is distinct from *Ar. pseudorasikravindrae,* which is a sister to *Ar. acutiapicum,* by the reduction of conidiophores to conidiogenous cells, cylindrical to ampulliform, pale brown conidiogenous cells with pointed, hyaline apex and brown to dark brown and smooth-walled conidia with a dark equatorial slit [34].

In our phylogenetic analysis based on combined LSU, ITS, TEF1-α and TUB2 sequence data, *Arthrinium acutiapicum* clustered with *Apiospora pseudorasikravindrae* (=*Ar. pseudorasikravindrae*) with high support (ML/BI = 95/-). Thus, we propose the transfer of *Ar. acutiapicum* under the new combination *Ap. acutiapica*, based on the morphological and phylogenetic analyses.

*Apiospora bambusicola* (X. Tang, K.D. Hyde and J.C. Kang) X.G. Tian and Tibpromma S., comb. nov.

Index Fungorum number: IF558493; Faces of Fungi number: FoF 09162.

Basioym: *Arthrinium bambusicola* X. Tang, K.D. Hyde and J.C. Kang, Biodiversity Data Journal 8 (e58755): 11 2020.

Notes: *Arthrinium bambusicola* was introduced by Tang et al. [18] and was collected on dead culms of *Schizostachyum brachycladum* in Thailand. Tang et al. [18] mentioned that *Ar. bambusicola* were retrieved as a sister taxon of *Ar. gutiae* with high support (ML/BI = 83/0.99), but differs from *Ar. gutiae* in having larger conidia and irregularly rounded, guttulate to roughened conidia. Pintos and Alvarado [4] transferred *Ar. gutiae* to *Apiospora* based on the phylogenetic analyses and morphological characters.

In our phylogenetic analyses based on combined LSU, ITS, TEF1-α and TUB2 sequence data, *Arthrinium bambusicola* is a sister to the newly introduced species *Ap. chiangraiense* with high support (ML/BI = 80/0.99). Thus, we propose the transfer of *Ar. bambusicola* under the new combination *Ap. bambusicola*, based on the morphological and phylogenetic analyses.

*Apiospora biserialis* (Y. Feng and Z.Y. Liu) X.G. Tian and Tibpromma S., comb. nov.

Index Fungorum number: IF558502; Faces of Fungi number: FoF 09569.

*Basioym: Arthrinium biseriale* Y. Feng, J.K. Liu, C.G. Lin, Y.Y. Chen, M.M. Xiang and Z.Y. Liu, *Frontiers in Microbiology* 12. 2021.

Notes: *Arthrinium biseriale* was introduced by Feng et al. [49] from dead culms of bamboo in China. The phylogenetic analysis of Feng et al. [49] showed that *Ar. biseriale* is closely related to *Ar. gelatinosum*, but they are phylogenetically distinct and can be recognized as two different species. Morphologically, *Ar. biseriale* has smaller stromata and the spores of *Ar. biseriale* are more curved than those of *Ar. gelatinosum* [49].

In our phylogenetic analyses based on combined LSU, ITS, TEF1-α and TUB2 sequence data, *Arthrinium biseriale* clustered with *Apiospora gelatinosa* with high support (ML/BI = 90/0.99). Thus, we propose the transfer of *Ar. biseriale* under the new combination *Ap. biserialis*, based on the morphological and phylogenetic analyses.

*Apiospora cordylines* (T.Z. Chen, Yong Wang bis and K.D. Hyde) X.G. Tian and Tibpromma S., comb. nov.

Index Fungorum number: IF558494.

*Basionym: Arthrinium cordylines* T.Z. Chen, Yong Wang bis and K.D. Hyde, *Mycotaxon* 136(1): 189 2021.

Notes: *Arthrinium cordylines* was introduced by Chen et al. [39] from the leaves of *Cordyline fruticosa* in China. Chen et al. [39] mentioned that *Ar. cordylinae* formed a well-supported branch with type strains of *Ar. aureum* (CBS 244.83) and *Ar. hydei* (CBS 114990). Meanwhile, a base difference comparison also confirmed *Ar. cordylinae* is a distinct species.

In our phylogenetic analyses, *Arthrinium cordylines* is a sister to *Ap. hydei* with high support (ML/BI = 96/0.99). Thus, we propose the transfer of *Ar. cordylines* under the new combination *Ap. cordylines*.

*Apiospora cyclobalanopsidis* (Y. Feng and Z.Y. Liu) X.G. Tian and Tibpromma S., comb. nov.

Index Fungorum number: IF558503; Faces of Fungi number: FoF 09572.

*Basioym: Arthrinium cyclobalanopsidis* Y. Feng, J.K. Liu, C.G. Lin, Y.Y. Chen, M.M. Xiang and Z.Y. Liu, *Frontiers in Microbiology* 12. 2021.

Notes: *Arthrinium cyclobalanopsidis* was introduced by Feng et al. [49] from a leaf of *Cyclobalanopsidis glauca* Oerst in China. Feng et al. [49] showed that *Ar. cyclobalanopsidis* clustered with *Ar. camelliae-sinensis*, but can be distinguished from *Ar. camelliae-sinensis* by conidiogenous cells. Pintos and Alvarado [4] transferred *Ar. camelliae-sinensis* to *Apiospora camelliae-sinensis*, based on the phylogenetic analyses and morphological characteristics.

In our phylogenetic analyses based on combined LSU, ITS, TEF1-α and TUB2 sequence data, *Arthrinium cyclobalanopsidis* clustered with *Ap. camelliae-sinensis* with high support (ML/BI = 78/1.00). Thus, we propose the transfer of *Ar. cyclobalanopsidis* under the new combination *Ap. cyclobalanopsidis*, based on the morphological and phylogenetic analyses.

*Apiospora euphorbiae* (M.B. Ellis) X.G. Tian and Tibpromma S., comb. nov.

Index Fungorum number: IF558495.

Basioym: *Arthrinium euphorbiae* M.B. Ellis, Mycol. Pap. 103: 6 1965.

Notes: *Arthrinium euphorbiae* was introduced by Ellis et al. [24] from the dead stems of *Euphorbia* in Zambia. Tang et al. [18] showed that *Ar. euphorbiae* is phylogenetically closely related to *Ar. malaysianum*, *Ar. vietnamensis*, and *Ar. chromolaenae* [4,18].

In our phylogenetic analyses, *Ar. euphorbiae* is a sister to *Ap. malaysiana* (=*Ar. malaysianum*) with strong bootstrap support values (ML/PP = 94/0.99). Thus, we propose the transfer of *Ar. euphorbiae* under the new combination *Ap. euphorbiae*.

*Apiospora gelatinosa* (Y. Feng and Z.Y. Liu) X.G. Tian and Tibpromma S., comb. nov.

Index Fungorum number: IF558504; Faces of Fungi number: FoF 09570.

*Basioym: Arthrinium gelatinosum* Y. Feng, J.K. Liu, C.G. Lin, Y.Y. Chen, M.M. Xiang and Z.Y. Liu, *Frontiers in Microbiology* 12. 2021.

Notes: *Arthrinium gelatinosum* was introduced by Feng et al. [49] from dead culms of bamboo in China. Feng et al. [49] mentioned that *Ar. gelatinosum* is a sister to *Ar. biseriale* with a well-supported lineage (ML/MP/BI = 93/98/1.00) [49].

In our phylogenetic analyses, *Arthrinium gelatinosum* clustered with *Apiospora biserialis* with high support (ML/BI = 90/0.99). Thus, we propose the transfer of *Ar. gelatinosum* under the new combination *Ap. gelatinosa*.

*Apiospora locuta*-*pollinis* (F. Liu and L. Cai) Pintos and P. Alvarado), X.G. Tian and Tibpromma S., comb. nov.

Index Fungorum number: 834523; Faces of Fungi number: FoF05221.

*Synonyms*: *Arthrinium pseudomarii* T.Z. Chen, Yong Wang bis and K.D. Hyde, *Mycotaxon* 136(1): 189. 2021.

*Basionym*: *Arthrinium locutum-pollinis* F. Liu and L. Cai (as ‘locuta- pollinis’), Mycosphere 9: 1094. 2018.

Notes: *Arthrinium pseudomarii* was introduced by Chen et al. [39] from the leaves of *Aristolochia debilis* in China. Chen et al. [39] mentioned that *Ar. pseudomarii* differs from *Ar. hispanicum, Ar. marii* and *Ar. mediterranei* by larger, subglobose to ellipsoid conidia and showed a close relationship with three species with high bootstrap support values (ML/MP = 95/93) [39].

In our phylogenetic analyses, *Ar. pseudomarii* (GUCC 10228) is a sister to *Ap. locuta-pollinis* (=*Ar. locuta-pollinis*) with high support of 95% ML. Based on the nucleotide comparisons, *Ar. pseudomarii* is slightly different from *Ap. locuta-pollinis* in 10/582 bp (1.72%) of ITS, but no base pair differences were observed in TUB2 and TEF1-α. Morphologically, the conidia of *Ar. pseudomarii* are similar to the holotype *Ap. locuta-pollinis* (LC 11683) in having similar size, brown with a hyaline equatorial rim, smooth, subglobose to ellipsoid condia and hyaline to pale brown, smooth, ampulliform to doliiform conidiogenous cells aggregated into clusters on the hyphae. Thus, we identified that they are the same species, and we synonymize *Ar. pseudomarii* under the *Ap. locuta-pollinis*, based on the morphological and phylogenetic analyses.

*Apiospora minutispora* (K. Das, S.Y. Lee and H.Y. Jung) X.G. Tian and Tibpromma S., comb. nov.

Index Fungorum number: IF558497.

*Basionym: Arthrinium minutisporum* K. Das, S.Y. Lee and H.Y. Jung, *Mycobiology* 48(6): 453 2020.

Notes: *Arthrinium minutisporum* was introduced by Das et al. [37] from mountain soil in Korea. Morphologically, *Ar. minutisporum* is quite similar to *Ar. phragmites, Ar. aureum* and *Ar. Hydei*. However, the conidia of *Ar. minutisporum* are smaller than those of *Ar. phragmites, Ar. aureum* and *Ar. Hydei,* and *Ar. minutisporum* produce smaller conidiogenous cells than *Ar. phragmites* [39]. Pintos and Alvarado [4] transferred *Ar. phragmites, Ar. aureum* and *Ar. hydei* to *Apiospora phragmites, Ap. aureum* and *Ap. hydei*, based on the phylogenetic analyses and morphological characteristics. Whereas *Ar. minutisporum* was maintained in *Arthrinium*.

In our phylogenetic analyses, *Arthrinium minutisporum* forms a distinct subclade and is close to *Apiospora aurea, Ap. balearica* and *Ap. descalsii* with strong bootstrap support values (ML/PP = 99/1.00) within *Apiospora*. Thus, we propose the transfer of *Ar. minutisporum* under the new combination *Ap. minutispora*.

*Apiospora pseudorasikravindrae* (Senan. and Cheew) X.G. Tian and Tibpromma S., comb. nov.

Index Fungorum number: IF 558505.

Basionym: *Arthrinium pseudorasikravindrae* Senan. and Cheew, Frontiers in Microbiology 11. 2020.

Notes: Arthrinium pseudorasikravindrae was introduced by Senanayake et al. [34] from dead twigs of Bambusa bambos Voss. in China. Arthrinium pseudorasikravindrae is morphologically distinct from Ar. chinense, Ar. paraphaeospermum and Ar. rasikravindrae by its thick-walled, finely roughened conidia with a pale, equatorial slit and ampulliform, cylindrical or doliiform, basauxic conidiogenous cells [34]. Pintos and Alvarado [4] transferred Ar. chinense, Ar. paraphaeospermum and Ar. rasikravindrae to Apiospora and synonymized them under Apiospora chinense, Ap. paraphaeospermum and Ap rasikravindrae, respectively, based on the phylogenetic analyses and morphological characteristics.

Our phylogenetic analyses based on combined LSU, ITS, TEF1-α and TUB2 sequence data show *Ar. pseudorasikravindrae* is a sister to the new combinations *Ap. acutiapica* *(=Ar. acutiapicum)* with high support (ML/BI = 77/0.99). Thus, we propose the transfer of *Ar. pseudorasikravindrae* under the new combination *Ap. pseudorasikravindrae*.

*Apiospora septata* (Y. Feng and Jian K. Liu) X.G. Tian and Tibpromma S., comb. nov.

Index Fungorum number: IF558506; Faces of Fungi number: FoF 09571.

*Basioym: Arthrinium septatum* Y. Feng, J.K. Liu, C.G. Lin, Y.Y. Chen, M.M. Xiang and Z.Y. Liu, *Frontiers in Microbiology* 12. 2021.

Notes: *Arthrinium septatum* was introduced by Feng et al. [49] from dead bamboo culms in China. Feng et al. [49] showed that *Arthrinium septatum* forms a well-supported clade and appears to be distinct from other *Arthrinium* species. *Arthrinium* *septatum* resembles *Ar. biseriale* in having a biseriate, broad fusiform to cylindrical ascospores and cylindrical, clavate asci. However, *Ar. septatum* differs from *Ar. biseriale* by having smaller stromata [49].

In our phylogenetic analyses, *Arthrinium septatum* groups in a well-supported clade with *Ap. pseudospegazzinii* and *Ap. gelatinosa*. Thus, we propose the transfer of *Ar. septatum* under the new combination *Ap. septata*, based on the morphological and phylogenetic analyses.

*Apiosporasetariae* (Jiang, N.; Tian, C.M.) X.G. Tian and Tibpromma S., comb. nov.

Index Fungorum number: IF835609.

Basioym: *Arthrinium setariae* JIANG, N.; TIAN, C.M. *Phytotaxa* 483, 149-159. 2021.

Notes: *Arthrinium setariae* was introduced by Jing et al. [38] from *Setaria viridis* in China. Jing et al. [38] mentioned that this species has larger conidia and is phylogenetically closely related to *Ar. jiangxiense*. Pintos and Alvarado [4] transferred *Ar. jiangxiense* to *Apiospora* and synonymized *Ap. jiangxiens* based on the phylogenetic analyses and morphological characteristics.

In our phylogenetic analyses based on combined LSU, ITS, TEF1-α and TUB2 sequence data, *Arthrinium setariae* clustered with *Apiospora jiangxiense* with high support (ML/BI = 87/1.00). Thus, we propose the transfer of *Ar. setariae* under the new combination *Ap. setariae*, based on the morphological and phylogenetic analyses.

*Apiospora sorghi* (J.D.P. Bezerra, C.M Gonçalves and C.M. Souza-Motta) X.G. Tian and Tibpromma S., comb. nov.

Index Fungorum number: IF558498; Faces of Fungi number: FoF 05762.

*Basioym*: *Arthrinium sorghi* J.D.P. Bezerra, C.M Gonçalves and C.M. Souza-Motta, *Fungal Diversity*: 10.1007, 73 2020.

Notes: *Arthrinium sorghi* was introduced as an endophyte by Bezerra et al. [36] from the leaves of *Sorghum bicolor* in Brazil. Bezerra et al. [36] mentioned that *Ar. sorghi* is treated as a unique lineage within *Arthrinium* based on ITS phylogenetic analysis. Morphologically, *Ar. sorghi* resembles *Ar. pseudosinense, Ar. ovatum* and *Ar. phaeospermum*, but differs from them by the culture characteristics, conidiophores and conidia size [36]. Pintos and Alvarado [4] transferred *Ar. pseudosinense, Ar. ovatum* and *Ar. phaeospermum* to *Apiospora pseudosinensis*, *Ap. ovata* and *Ap. phaeospermum* based on the phylogenetic analyses and morphological characteristics.

In our phylogenetic analyses based on combined LSU, ITS, TEF1-α and TUB2 sequence data, *Arthrinium sorghi* clustered with *Apiospora bambusucila* with high support (ML/BI = 78/0.99). Thus, we propose the transfer of *Ar. sorghi* under the new combination *Ap. sorghi*, based on the morphological and phylogenetic analyses.

## 4. Discussion

In this study, the new taxon *Apiospora chiangraiense* and two new host records, viz., *Ap. intestini* and *Ap. rasikravindrae,* are introduced based on the morphological and phylogenetic analyses. In addition, thirteen new combinations are proposed based on the phylogenetic analyses.

*Apiospora* was previously synonymized under *Arthrinium*, but Pintos et al. [14] re-evaluated the placements of these two genera and transferred 55 species to *Apiospora* based on a phylogenetic analysis. Currently, 117 species of *Apiospora* are listed in the Index Fungorum [33]. Among these 117 species, 55 species have been confirmed in *Apiospora* by phylogenetic analyses [4]; however, the remaining 62 species need to be confirmed, as the sequence data of these species are not available. The morphology of *Apiospora* and *Arthrinium* are quite similar, so it is difficult to distinguish *Apiospora* and *Arthrinium* based only on morphology.

In the phylogenetic analyses, two *Arthrinium* species, viz., *Arthrinium trachycarpum* and *Ar. urticae,* formed a distinct clade out of *Arthrinium*, and this result is consistent with previous studies [18]. However, the morphologies of these two species are not significantly different from *Arthrinium*; thus, more collections are required to clarify the placement of these two species [24,50]. In addition, our phylogenetic analyses showed that *Apiospora sorghi*, *Ap. bambusucila*, *Ap. chiangraiense* and *Ap. intesini* are not clustered together in *Apiospora* major clades (Figure 1). We also compared the LSU sequence of these four species with other *Apiospora* species, but a few base pair differences were found. Moreover, their morphologies fit well within the species concept of *Apiospora*. Thus, further phylogenetic analyses are necessary to confirm whether *Apiospora* is a species complex or not.

## Figures and Tables

**Table 1:** Taxa names, strain numbers, host, countries and corresponding GenBank accession numbers of the taxa used in the phylogenetic analyses of this study.

Taxa Names	Strain Numbers	Substrates	Countries	GenBank Accession Numbers			
				ITS	LSU	TUB2	TEF 1-α
*Apiospora acutiapica*	KUMCC 20-0210	*Bambusa bambos*	China	MT946343	MT946339	MT947366	MT947360
*Apiospora aquaticum*	S-642	Submerged wood	China	MK828608	MK835806	-	-
*Apiospora arundinis*	CBS 133509	*Aspergillus flavus* sclerotium Buried in sandy field	USA	KF144886	KF144930	KF144976	KF145018
*Apiospora arundinis*	CBS 449.92	Bamboo	Canada	KF144887	KF144931	KF144977	KF145019
*Apiospora aurea*	**CBS 244.83**	-	Japan	AB220251	KF144935	KF144981	KF145023
*Apiospora balearica*	**CBS 145129**	Undetermined poaceae	Spain	MK014869	MK014836	MK017975	MK017946
*Apiospora bambusae*	**ICPM 6889**	Bamboo	New Zealand	MK014874	MK014841	MK017980	MK017951
*Apiospora bambusae*	CBS 145133	*Phyllostachys aurea*	Spain	MK014875	MK014842	MK017981	MK017952
*Apiospora bambusicola*	**MFLUCC20-0144**	*Schizostachyum brachycladum*	Thailand	MW173030	MW173087	-	MW183262
*Apiospora biserialis*	**CGMCC 3.20135**	**Bamboo**	**China**	**MW481708**	**MW478885**	**MW522955**	**MW522938**
*Apiospora biserialis*	GZCC 20_0099	Bamboo	China	MW481709	MW478886	MW522956	MW522939
*Apiospora biserialis*	GZCC 20_0100	Bamboo	China	MW481710	MW478887	MW522957	MW522940
*Apiospora camelliae-sinensis*	**LC 5007**	*Camellia sinensis*	China	KY494704	KY494780	KY705173	KY705103
*Apiospora camelliae-sinensis*	LC 8181	*Brassica rapa*	China	KY494761	KY494837	KY705229	KY705157
*Apiospora chromolaenae*	**MFLUCC17-1505**	*Chromolaena odorata*	Thailand	MT214342	MT214436	-	MT235802
*Apiospora chiangraiense* ^▲^	**MFLUCC21-0053**	Dead culms of bamboo	Thailand	MZ542520	MZ542524	MZ546409	-
*Apiospora cordylinae*	GUCC 10026	*Cordyline fruticosa*	China	MT040105	-	MT040147	MT040126
*Apiospora cyclobalanopsidis*	**CGMCC 3.20136**	*Cyclobalanopsidis glauca*	**China**	**MW481713**	**MW478892**	**MW522962**	**MW522945**
*Apiospora cyclobalanopsidis*	GZCC 20_0103	*Cyclobalanopsidis glauca*	China	MW481714	MW478893	MW522963	MW522946
*Apiospora descalsii*	**CBS 145130**	*Ampelodesmos mauritanicus*	Spain	MK014870	MK014837	MK017976	MK017947
*Apiospora dichotomanthi*	**LC 4950**	*Dichotomanthes tristaniicarpa*	China	KY494697	KY494773	KY705167	KY705096
*Apiospora dichotomanthi*	LC 8175	*Dichotomanthes tristaniicarpa*	China	KY494755	KY494831	KY705223	KY705151
*Apiospora esporlensis*	**CBS 145136**	*Phyllostachys aurea*	Spain	MK014878	MK014845	MK017983	MK017954
*Apiospora euphorbiae*	IMI 285638b	*Bambusa* sp.	Bangladesh	AB220241	AB220335	AB220288	-
*Apiospora gaoyouensis*	**CFCC 52301**	*Phragmites australis*	China	MH197124	-	MH236789	MH236793
*Apiospora gaoyouensis*	CFCC 52302	*Phragmites australis*	China	MH197125	-	MH236790	MH236794
*Apiospora garethjonesii*	**KUMCC 16-0202**	Dead culms of bamboo	China	KY356086	KY356091	-	-
*Apiospora gelatinosa*	**KHAS 11962**	**Bamboo**	**China**	**MW481706**	**MW478888**	**MW522958**	**MW522941**
*Apiospora gelatinosa*	GZAAS 20-0107	Bamboo	China	MW481707	MW478889	MW522959	MW522942
*Apiospora guizhouensis*	LC 5318	Air in karst cave	China	KY494708	KY494784	KY705177	KY705107
*Apiospora guizhouensis*	**LC 5322**	Air in karst cave	China	KY494709	KY494785	KY705178	KY705108
*Apiospora hispanica*	**IMI 326877**	Beach sand	Spain	AB220242	AB220336	AB220289	-
*Apiospora hydei*	**CBS 114990**	*Bambusa tuldoides*	China	KF144890	KF144936	KF144982	KF145024
*Apiospora hydei*	KUMCC 16-0204	Dead culms of bamboo	China	KY356087	KY356092	-	-
*Apiospora hyphopodii*	**MFLUCC15-0003**	*Bambusa tuldoides*	China	KR069110	-	-	-
*Apiospora hyphopodii*	KUMCC 16-0201	Culms of bamboo	China	KY356088	KY356093	-	-
*Apiospora iberica*	**CBS 145137**	*Arundo donax*	Portugal	MK014879	MK014846	MK017984	MK017955
*Apiospora intestini*	**CBS 135835**	Gut of a grasshopper	India	KR011352	MH877577	KR011350	KR011351
*Apiospora intestini* ^▲^	MFLUCC 21-0052	Dead culms of bamboo	Thailand	MZ542521	MZ542525	MZ546410	MZ546406
*Apiospora italica*	**CBS 145138**	*Arundo donax*	Italy	MK014880	MK014847	MK017985	MK017956
*Apiospora italica*	CBS 145139	*Phragmites australis*	Spain	MK014881	MK014848	MK017986	-
*Apiospora jatrophae*	**AMH-9557**	*Jatropha podagrica*	India	JQ246355	-	-	-
*Apiospora jatrophae*	AMH-9556	*Jatropha podagrica*	India	HE981191	-	-	-
*Apiospora jiangxiensis*	LC 4494	*Phyllostachys* sp.	China	KY494690	KY494766	KY705160	KY705089
*Apiospora jiangxiensis*	**LC 4577**	*Maesa* sp.	China	KY494693	KY494769	KY705163	KY705092
*Apiospora kogelbergensis*	CBS 113332	*Cannomois virgata*	South Africa	KF144891	KF144937	KF144983	KF145025
*Apiospora kogelbergensis*	**CBS 113333**	Dead culms of Restionaceae	South Africa	KF144892	KF144938	KF144984	KF145026
*Apiospora locuta* *-pollinis*	LC 11688	Bee bread	China	MF939596	-	MF939623	MF939618
*Apiospora locuta* *-pollinis*	**LC 11683**	*Brassica campestris*	China	MF939595	-	MF939622	MF939616
*Apiospora longistroma*	MFLUCC 11-0479	Dead culms of bamboo	Thailand	KU940142	KU863130	-	-
*Apiospora longistroma*	**MFLUCC11-0481**	Dead culms of bamboo	Thailand	KU940141	KU863129	-	-
*Apiospora malaysiana*	**CBS 102053**	*Macaranga hullettii*	Malaysia	KF144896	KF144942	KF144988	KF145030
*Apiospora marii*	**CBS 497.90**	Beach sands	Spain	AB220252	KF144947	KF144993	KF145035
*Apiospora marii*	DiSSPA_A1	Oleaeuropaea	Italy	MK602320	-	MK614695	MK645472
*Apiospora mediterranea*	**IMI 326875**	Air	Spain	AB220243	AB220337	AB220290	-
*Apiospora minutispora*	1.70E-41	Mountain soil	Korea	LC517882	-	LC518888	LC518889
*Apiospora mytilomorpha*	**DAOM 214595**	*Andropogon* sp.	India	KY494685	-	-	-
*Apiospora neobambusae*	**LC 7106**	Leaves of bamboo	China	KY494718	KY494794	KY705186	KY806204
*Apiospora neobambusae*	LC 7124	Leaves of bamboo	China	KY494727	KY494803	KY705195	KY806206
*Apiospora neochinensis*	**CFCC 53036**	*Fargesia qinlingensis*	China	MK819291	-	MK818547	MK818545
*Apiospora neochinensis*	CFCC 53037	*Fargesia qinlingensis*	China	MK819292	-	MK818548	MK818546
*Apiospora neogarethjonesii*	**DQD 2019a**	Bamboo	China	MK070897	MK070898	-	-
*Apiospora neosubglobosa*	JHB 006	Bamboo	China	KY356089	KY356094	-	-
*Apiospora neosubglobosa*	**KUMCC 16-0203**	Bamboo	China	KY356090	KY356095	-	-
*Apiospora obovata*	**LC 4940**	*Lithocarpus* sp.	China	KY494696	KY494772	KY705166	KY705095
*Apiospora obovata*	LC 8177	*Lithocarpus* sp.	China	KY494757	KY494833	KY705225	KY705153
*Apiospora ovata*	**CBS 115042**	*Arundinaria hindsii*	China	KF144903	KF144950	KF144995	KF145037
*Apiospora paraphaeosperma*	**MFLUCC13-0644**	Dead culms of bamboo	Thailand	KX822128	KX822124	-	-
*Apiospora phragmitis*	**CPC 18900**	*Phragmites australis*	Italy	KF144909	KF144956	KF145001	KF145043
*Apiospora phyllostachydis*	**MFLUCC18-1101**	*Phyllostachys heteroclada*	China	MK351842	MH368077	MK291949	MK340918
*Apiospora piptatheri*	**CBS 145149**	*Piptatherum miliaceum*	Spain	MK014893	MK014860	-	MK017969
*Apiospora pseudomarii*	**GUCC 10228**	*Aristolochia debilis*	China	MT040124	-	MT040166	MT040145
*Apiospora pseudoparenchymatica*	**LC 7234**	Leaves of bamboo	China	KY494743	KY494819	KY705211	KY705139
*Apiospora pseudoparenchymatica*	LC 8173	Leaves of bamboo	China	KY494753	KY494829	KY705221	KY705149
*Apiospora pseudorasikravindrae*	**KUMCC 20-0208**	*Bambusa dolichoclada*	China	MT946344	-	MT947367	MT947361
*Apiospora pseudosinensis*	**CPC 21546**	Leaves of bamboo	Netherlands	KF144910	KF144957	-	KF145044
*Apiospora pseudospegazzinii*	**CBS 102052**	*Macaranga hullettii*	Malaysia	KF144911	KF144958	KF145002	KF145045
*Apiospora pterosperma*	CBS 123185	*Machaerina sinclairii*	New Zealand	KF144912	KF144959	KF145003	-
*Apiospora pterosperma*	**CPC 20193**	*Lepidosperma gladiatum*	Australia	KF144913	KF144960	KF145004	KF145046
*Apiospora qinlingensis*	**CFCC 52303**	Fargesiaqinlingensis	China	MH197120	-	MH236791	MH236795
*Apiospora qinlingensis*	CFCC 52304	*Fargesia qinlingensis*	China	MH197121	-	MH236792	MH236796
*Apiospora rasikravindrae*	LC 8179	*Brassica rapa*	China	KY494759	KY494835	KY705227	KY705155
*Apiospora rasikravindrae*	**NFCCI 2144**	Soil	Norway	JF326454	-	-	-
*Apiospora rasikravindrae* ^▲^	MFLUCC 21-0051	Dead culms of bamboo	Thailand	MZ542523	MZ542527	MZ546412	MZ546408
*Apiospora rasikravindrae* ^▲^	MFLUCC 21-0054	Dead culms of *Maize*	Thailand	MZ542522	MZ542526	MZ546411	MZ546407
*Apiospora sacchari*	CBS 372.67	Air	-	KF144918	KF144964	KF145007	KF145049
*Apiospora sacchari*	CBS 664.74	Soil under *Calluna vulgaris*	Netherlands	KF144919	KF144965	KF145008	KF145050
*Apiospora saccharicola*	CBS 191.73	Air	Netherlands	KF144920	KF144966	KF145009	KF145051
*Apiospora saccharicola*	CBS 831.71	-	Netherlands	KF144922	KF144969	KF145012	KF145054
*Apiospora septata*	**CGMCC 3.20134**	**bamboo**	**China**	**MW481711**	**MW478890**	**MW522960**	**MW522943**
*Apiospora septata*	GZCC 20_0109	bamboo	China	MW481712	MW478891	MW522961	MW522944
*Apiospora serenensis*	**IMI 326869**	Food, pharmaceutical excipients, atmosphere and home dust	Spain	AB220250	AB220344	AB220297	-
*Apiospora setariae*	MT492005	*Setaria viridis*	China	MT492005	-	MT497467	MW118457
*Apiospora setostroma*	**KUMCC 19-0217**	Dead branches of bamboo	China	MN528012	MN528011	-	MN527357
*Apiospora sorghi*	**URM 93000**	*Sorghum bicolor*	Brazil	MK371706	-	MK348526	-
*Apiospora subglobosa*	**MFLUCC11-0397**	Dead culms of bamboo	Thailand	KR069112	KR069113	-	-
*Apiospora subrosea*	LC 7291	Leaves of bamboo	China	KY494751	KY494827	KY705219	KY705147
*Apiospora subrosea*	**LC 7292**	Leaves of bamboo	China	KY494752	KY494828	KY705220	KY705148
*Apiospora thailandica*	MFLUCC 15-0199	Dead culms of bamboo	Thailand	KU940146	KU863134	-	-
*Apiospora thailandica*	**MFLUCC15-0202**	Dead culms of bamboo	Thailand	KU940145	KU863133	-	-
*Apiospora vietnamensis*	**IMI 99670**	Citrus sinensis	Vietnam	KX986096	KX986111	KY019466	-
*Apiospora xenocordella*	**CBS 478.86**	Soil from roadway	Zimbabwe	KF144925	KF144970	KF145013	KF145055
*Apiospora xenocordella*	CBS 595.66	Soil	Austria	KF144926	KF144971	-	-
*Apiospora yunnana*	DDQ 00281	*Phyllostachys nigra*	China	KU940148	KU863136	-	-
*Apiospora yunnana*	**MFLUCC15-1002**	*Phyllostachys nigra*	China	KU940147	KU863135	-	-
*Arthrinium austriacum*	GZU 345004	*Carex pendula*	Austria	MW208928	-	-	-
*Arthrinium austriacum*	GZU 345006	*Carex pendula*	Austria	MW208929	MW208860	-	-
*Arthrinium* cf. *sporophleoides*	GZU 345102	Carex firma	Austria	MW208944	MW208866	-	-
*Arthrinium caricicola*	CBS 145127	*Carex ericetorum*	China	MK014871	MK014838	MK017977	MK017948
*Arthrinium crenatum*	**AG 19066**	*Poaceae*, *Carex*	France	MW208931	MW208861	-	-
*Arthrinium curvatum*	AP 25418	Leaves of *Carex* sp.	China	MK014872	MK014839	MK017978	MK017949
*Arthrinium japonicum*	IFO 30500	-	Japan	AB220262	AB220356	AB220309	-
*Arthrinium japonicum*	IFO 31098	Leaves of *Carex despalata*	Japan	AB220264	AB220358	AB220311	-
*Arthrinium luzulae*	**AP7619-3**	*Luzula sylvatica*	Spain	MW208937	MW208863	-	-
*Arthrinium morthieri*	GZU 345043	*Cyperaceae carex*	Austria	MW208938	MW208864	-	-
*Arthrinium phaeospermum*	CBS 114317	Leaves of *Hordeum vulgare*	Iran	KF144906	KF144953	KF144998	KF145040
*Arthrinium phaeospermum*	CBS 114318	Leaves of *Hordeum vulgare*	Iran	KF144907	KF144954	KF144999	KF145041
*Arthrinium puccinioides*	CBS 549.86	*Lepidosperma gladiatum*	Germany	AB220253	AB220347	AB220300	-
*Arthrinium sphaerospermum*	AP25619/CBS 146355	*Poaceae*	Norway	MW208943	MW208865	-	-
*Arthrinium sporophleum*	CBS 145154	*Dead leaves of Juncus* sp.	Spain	MK014898	MK014865	MK018001	MK017973
*Arthrinium trachycarpum*	**CFCC 53038**	*Trachycarpus fortune*	China	MK301098	-	MK303394	MK303396
*Arthrinium urticae*	IMI 326344	*-*	-	AB220245	AB220339	AB220292	-
*Arthrinium trachycarpum*	CFCC 53039	*Trachycarpus fortune*	China	MK301099	-	MK303395	MK303397
*Nigrospora aurantiaca*	**CGMCC 3.18130**	*Nelumbo* sp.	China	KX986064	KX986098	KY019465	KY019295
*Nigrospora camelliae* *-sinensis*	**CGMCC 3.18125**	*Camellia sinensis*	China	KX985986	KX986103	KY019460	KY019293
*Nigrospora chinensis*	**CGMCC 3.18127**	*Machilus breviflora*	China	KX986023	KX986107	KY019462	KY019422
*Nigrospora gorlenkoana*	CBS 480.73	*Vitis vinifera*	Kazakhstan	KX986048	KX986109	KY019456	KY019420
*Nigrospora guilinensis*	**CGMCC 3.18124**	*Camellia sinensis*	China	KX985983	KX986113	KY019459	KY019292
*Nigrospora hainanensis*	**CGMCC 3.18129**	*Musa paradisiaca*	China	KX986091	KX986112	KY019464	KY019415
*Nigrospora lacticolonia*	**CGMCC 3.18123**	*Camellia sinensis*	China	KX985978	KX986105	KY019458	KY019291
*Nigrospora musae*	CBS 319.34	*Musa* sp.	Australia	MH855545	KX986110	KY019455	KY019419
*Nigrospora oryzae*	LC2693	*Neolitsea* sp.	China	KX985944	KX986101	KY019471	KY019299
*Nigrospora osmanthi*	**CGMCC 3.18126**	*Hedera nepalensis*	China	KX986010	KX986106	KY019461	KY019421
*Nigrospora pyriformis*	**CGMCC 3.18122**	*Citrus sinensis*	China	KX985940	KX986100	KY019457	KY019290
*Nigrospora rubi*	**LC2698**	*Rubus* sp.	China	KX985948	KX986102	KY019475	KY019302
*Nigrospora sphaerica*	LC7298	*Nelumbo* sp.	China	KX985937	KX986097	KY019606	KY019401
*Nigrospora vesicularis*	**CGMCC 3.18128**	*Musa paradisiaca*	China	KX986088	KX986099	KY019463	KY019294
*Sporocadus trimorphus*	**CBS 114203**	*Rosa canina*	Sweden	MH553977	MH554196	MH554636	MH554395

Notes: Newly generated sequences are indicated by ▲ after the species name. Ex-type strains are in bold. - = information not available. Abbreviations: AMH: Ajrekar Mycological Herbarium, Pune, Maharashtra, India; CBS: Westerdijk Fungal Biodiversity Institute, Utrecht, Netherlands; CFCC: China Forestry Culture Collection Center, Beijing, China; CPC: Culture collection of Pedro Crous, housed at the Westerdijk Fungal Biodiversity Institute; DAOM: Canadian Collection of Fungal Cultures, Ottawa, Canada; DDQ: D.Q. Dai; GUCC: Guizhou University Culture Collection, Guizhou, China; ICMP: International Collection of Microorganisms from Plants, New Zealand; IFO: Institute for Fermentation, Osaka, Japan; IMI: Culture collection of CABI Europe UK Centre, Egham, UK; JHB: H.B. Jiang; KUMCC: Culture collection of Kunming Institute of Botany, Yunnan, China; LC: Personal culture collection of Lei Cai, housed in the Institute of Microbiology, Chinese Academy of Sciences, China; MFLUCC: Mae Fah Luang University Culture Collection, Chiang Rai, Thailand; NFCCI: National Fungal Culture Collection of India.

## Data Availability

Not applicable.
